# Community knowledge, attitudes, and practices regarding the use of plants for mosquito control: The case of Arjo Gudatu District, East Wollega Zone, Oromia Regional State, Ethiopia

**DOI:** 10.1016/j.parepi.2025.e00463

**Published:** 2025-11-04

**Authors:** Lensa Tesfaye, Esayas Aklilu, Ketema Tolossa, Abebe Animut

**Affiliations:** Aklilu Lemma Institute of Health Science, Addis Ababa University, Ethiopia

**Keywords:** Arjo Gudatu, Ethiopia, Knowledge, Mosquitoes, Plant

## Abstract

**Background:**

Mosquitoes are the species that transmit malaria and various viral diseases to humans. The main mosquito control strategies, particularly indoor spraying with insecticides and long-lasting insecticide-treated nets, have proven to reduce the incidence and spread of malaria. However, their effectiveness is threatened by the emergence and spread of insecticide-resistant vectors. Indigenous plants offer a safe, effective, and locally accessible solution to address the rising threat of mosquito-borne diseases.

**Objective:**

To assess the knowledge, attitudes, and practices of the inhabitants of Arjo Gudatu District, Ethiopia, regarding the use of plants to control mosquitoes.

**Materials and methods:**

A descriptive cross-sectional study was conducted among the inhabitants of Mada Jalala, Karsa Dako, and Lalisa Dimtu kebeles (the lowest administrative level) in the Arjo Gudatu district, Oromia Regional State, Ethiopia. Randomly selected households were interviewed using a questionnaire developed for this study. The data were collected through door-to-door interviews, checked for completeness and consistency, entered into SPSS version 25, cleaned, and analyzed.

**Results:**

Of the 398 household representatives interviewed, 63.5 % (253 individuals) reported knowing the use of plants for mosquito control. Of these, about 75 % (190 out of 253) plants were for this purpose, and 79 % (200 out of 253) expressed a positive attitude to using plants to control mosquitoes. The most frequently mentioned plant was *Securidaca Longpedunculata* (31 respondents, 12 %), followed by *Echinops Sphaocephalus L.* (28 respondents, 11 %) and *Agave Sisalana Perrine Ex Engelm.* (21 respondents, 8.3 %). These plants were cited for their roles as repellents, adulticides, and larvicides.

**Conclusion:**

*Agave sisalana Perrine ex Engeln, Securidaca longepedunculate,* and *Echinops sphaerocephalus* L. were the plants most commonly used to control mosquitoes. The plants were used in traditional mosquito control as adulticides, larvicides, and repellents.

## 1 Background

Arthropods such as mosquitoes, ticks, and sandflies pose the greatest health and economic problems worldwide (Thomson, 2014). They keep over 3.9 billion people at risk of infection and death (Kweka et al., 2008). Mosquito comprise the species that transmit malaria, filariasis, Japanese encephalitis, dengue fever, yellow fever, West Nile fever, and chikungunya, among others (WHO, 2020). Every year, mosquito-transmitted diseases infect approximately 700 million people and cause more than one million deaths (Dilani et al., 2022). In Ethiopia, *Anopheles arabiensis*, *Anopheles funestus*, *Anopheles pharoensis*, and *Anopheles Nili* transmit malaria of which *Anopheles arabiensis* is the predominant malaria vector (Federal ministry of health, 2016; Yewhalaw et al., 2010). *Aedes aegypti* also prevails in the country where temperatures are consistently above 10 °C (50 °F). It is the leading vector of arboviral diseases (Public Health Entomology Research Team, 2021).

Malaria-associated morbidity and mortality decreased dramatically in Ethiopia as a result of the use of indoor residual insecticide spraying (IRS) and long-lasting insecticide-treated mosquito nets (LLINs) (Taffese et al., 2018). Although these methods played a significant role in reducing malaria, their efficacy has been challenged for several reasons. First, *Anopheles arabiensis* has developed resistance to insecticides (Bedasso et al., 2022). Second, the methods fail to control vectors that exhibit exophagic/exophilic behavior and bite during the early hours of the night (Carnevale and Manguin, 2021). Furthermore, insecticides threaten the environment as well as human health (Ghosh, 1991).

These challenges rekindled a need for the development of alternative or complementary vector control approaches. Plants can serve as alternative insecticides and repellents due to their availability, ecological safety, minimal effect on non-target organisms, specificity against target pests, reduced likelihood of resistance, extended effectiveness requiring less frequent application, cultural acceptability, and suitability for rural settings (Ghosh, 1991; Bekele and Asfaw, n.d.). In addition, natural plants contain bioactive compounds that are active against target insects, biodegradable, active against target insects, and have been effectively used against mosquitoes. Currently, it is thought that using plant-based bio-insecticides is far safer and less harmful than synthetic bio-insecticides (Baluselvakumar et al., 2012). Plants are also ecologically safe, specific against target pests, less likely to be resistant, effective, culturally acceptable, and suitable for rural settings (Markouk et al., 2001). In Ethiopia, where malaria prevails, traditional healthcare practices have long relied on herbal remedies to control blood-sucking insects such as mosquitoes. For instance, Karunamoorthi and Hailu (2014) reported that *Cupressus lusitanica*, *Eucalyptus globulus*, and *Boswellia papyrifera* serve as mosquito repellents (Karunamoorthi and Hailu, 2014). In the Seweyna District of the Bale Zone, Southeast Ethiopia, approximately 19 plant species were used by the local community to repel mosquitoes. Among these, *Mimusops kummel* and *Acokanthera schimperi* were the most frequently mentioned (Shibeshi et al., 2024). *Allium sativum* (80.55 %), *Echinops Jericho* (75.11 %), *and Eucalyptus citriodora* (61.66 %) are used for malaria prevention in western regions of the country (Kenea, 2015). These underscore the potential of plants as alternative insecticides. To this end, documenting the traditional use of plants remains the basis for scientific evaluation of their mosquitocidal activity and analysis of bioactive compounds. This study assessed the knowledge, attitudes, and practices of members of the communities of Mada Jalala, Karsa Dako, and Lalisa Dimtu kebeles, Arjo Gudatu district, Ethiopia, regarding the use of plants to control mosquitoes.

## 2 Methods and materials

### 2.1 Study area and design

**Fig. 1:**
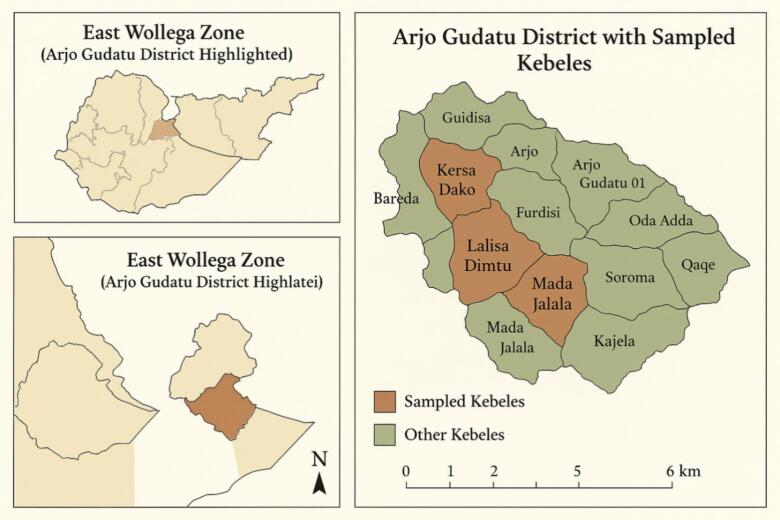
Map of Arjo Gudatu District, East Wollega Zone in the Oromia Regional State, Ethiopia. (Source: ARC map).

The study was conducted in the Arjo Gudatu district, Eastern Wollega Zone, Oromia Regional State, Ethiopia. The community-based cross-sectional study design was conducted from June 30 to September 25, 2023 (Fig. 1). The district is located 343 km southwest of the capital, Addis Ababa, and 42 km from the city of Nekemte. Its altitude is between 1100 and 2300 m above sea level. The district has an average annual rainfall of 1400 mm (1200–2000 mm) and an average annual temperature of 18 °C to 32 °C. According to the Central Statistics Agency (2021), the Oromo ethnic group makes up 98 % of the population. Maize, millet, and mango are grown in the district. The district is divided into fifteen rural kebeles (kebeles, the lowest administrative structure). Three of these kebeles (Mada Jalala, Karsa Dako, and Lalisa Dimtu) were selected for the study. The selected kebeles have a common health center, and each of them has a health post. In total, the district has one health center and 15 health posts. The selected kebeles are malarious in part due to their suitable ecology, which includes an abundance of small rivers and irrigated agriculture. In addition, the ongoing political unrest in the region may have exacerbated the transmission of the disease by affecting the health system (Arjo Gudatu Health Center unpublished data), possibly forcing local people to use indigenous plants to control mosquito bites.

### 2.2 Sample size calculation and sampling technique

The sample size was determined using the simple population proportion formula, which effectively measures the practice's prevalence in the community. The formula used was: n = Z^2^ (1-p)/d^2^ with a default rate of 50 %. Where Z corresponds to a 95 % confidence level and d = the margin of error, which was set at 5 % and a 5 % non-response rate. Consequently, the final sample size for this study was 403. The total sample size was divided proportionally based on the number of households in each kebele, relative to the total number of households. The total sample size was divided proportionally based on the number of households in each kebele, relative to the total number of households. This allocation was calculated using the formula for strata: nh = Nh/N × n.

Where:

• nh = sample size for stratum h
• Nh = population size in stratum h
• N = total population size
• n = total sample size

Accordingly, 150 households were selected from Mada Jalala, 144 from Lalisa Dimtu, and 104 from Kersa Dako. In each kebele, the first household was chosen from a household list, followed by systematic sampling: every 3rd household in Mada Jalala and Lalisa Dimtu, and every 4th household in Kersa Dako. Data were collected using a semi-structured questionnaire (see Appendix II for the complete list of questions). Interviews were conducted with household representatives aged 18 years and above. The questionnaire was initially developed in English, translated into the local language (Afan Oromo) to ensure clarity during interviews, and subsequently translated back into English for data entry, analysis, and interpretation.

#### 2.2.1 Collection of knowledge, attitudes, and practices data

During the survey, participants were first asked about their socio-demographic backgrounds, followed by questions assessing their knowledge, practices, and attitudes regarding the use of plants for mosquito control. Respondents identified the plant species they use to repel or kill insects, specified the insect types targeted, and provided details on each plant's growth form, origin (wild or cultivated), and local name. They further described the methods of preparation and application, as well as the specific plant parts used. For each plant mentioned, follow-up questions addressed its accessibility (ease of local acquisition) and affordability (perceived cost). Although data were gathered for all cited plant species, this study presents summarized results for only the most frequently mentioned plants. All responses summarized in Table 2, Table 3 are based solely on direct participant knowledge and experience, without reference to literature sources. Plant specimens were collected during field visits to various locations in Arjo Gudatu District, Oromia Regional State, Ethiopia, and were identified based on herbarium material, experts, and taxonomic keys in different volumes of Flora of Ethiopia (Hedberg et al., 2003; Hedberg et al., 2004). The voucher specimens were deposited in the National Herbarium of Addis Ababa University, Ethiopia, for future reference.

### 2.3 Data management and analysis

The data were checked for completeness and consistency, entered into SPSS version 25, cleaned, and analyzed. The results are presented as simple frequencies with percentages in tables and diagrams. Chi-square tests were used to evaluate associations between demographic variables (e.g., gender, age group, education level) and the frequency of plant citations, as well as to test for significant differences in reported plant use between study Mada Jalala, Karsa Dako, and Lalisa Dimtu kebeles. The level of significance was determined using 95 % confidence intervals (CIs) and the *p*-value. A simple preference ranking was used for the most frequently mentioned plants. Participants were asked to list their preferred plants for vector control, from highest to lowest of the twelve plants, to identify the plants most preferred by the communities.

### 2.4 Ethics statement

The study was approved by the Institutional Research Ethics Review Committee (IRERC) of the Aklilu Lemma Institute of Pathobiology (ALIPB), Addis Ababa College, Ethiopia (Ref. No. ALIPB IRERC/108/2015/23). Meetings were held with Arjo Gudatu Health Center, district leaders, and neighborhood association members before the survey started to explain the study's objectives. A written consent form was obtained from each participant before the survey. Each participant was assured that they could withdraw at any interview stage if they so wished. Identification numbers were used for the study instead of participants' names, and the information collected was kept confidential.

## 3 Results

### 3.1 Sociodemographic characteristics

Of the total of 403 expected respondents, 398 took part in the interview (a response rate of 98.7 %). Approximately 67.6 % (269) of the participants were male (Table 1). Their ages ranged from 25 to 75 years. Nearly 75 % (298) were farmers, and the average household size of respondents was 6.4 ± 2.1 members, slightly above the national average of 4.6 reported by the Ethiopian Demographic and Health Survey (EDHS, 2019). The average monthly income was 1350 Ethiopian Bir (ETB), 420, which is lower than the national average of approximately 2500 ETBs. More than half of those surveyed, 50.5 % (201), can neither read nor write.

**Table 1:** Socio-demographic characteristics of the household of Lalisa Dimtu, Karsa Dako, and Mada Jalala kebeles, Arjo Gudatu district, Oromia region, Ethiopia.

Variable	Category	Frequency (%)
Sex	Male	269 (67.6)
	Female	129 (32.4)
Age	19–30	21(5.3)
	31–40	61 (15.3)
	41–50	121(30.4)
	51–60	124 (31.2)
	>60	71(17.8)
Family size	1–2	16(4)
	3–4	62(15.6)
	5–6	114(28.6)
	>6	206(51.8)
Educational level	Illiterate	201(50.5)
	Read & write	75 (18.8)
	1–5	70(17.6)
	6–8	27(6.8)
	9–10	25(6.3)
Occupational	Farmer	298(74.9)
	Merchant	30(8)
	Civil servant	16(4)
	Housewife	54(13.6)
	Daily labor	27(6.8)
Income	<500	89 (22.4)
	501–700	63(15.8)
	701–900	67(16.8)
	901–1100	92 (23.1)
	>1100	87 (21.9)
Do you know Plants are used to control mosquitoes (*n* = 398)	Yes	253 (63.5 %)
	No	145(36.5 %)
Source of knowledge regarding plants used for mosquito control (*n* = 253)	Community	152 (60 %)
	Family	101(40 %)

### 3.2 Knowledge of the respondents on the use of plants to control mosquitoes

Overall, 63.6 % (253/398) perceived that plants are used to control mosquitoes, among which 39.9 % (101/253) heard from family and 60 % (152/253) from their community (Table 1). A total of 20 plant species were identified by the communities of the villages for mosquito control. About 60 % (12 species) of them were classified as herbs and 40 % (8 species) as trees (Table 2). The most frequently (12.3 %; 31/253) reported species was *Securidaea longepedunculate*, cited, followed by *Echinops sphaerocephalus L*. (11 %; 28/253), and *Agave sisalana Perrine ex Engeln.* (8.3 %; 21). Approximately 40 % (8/20) of the plants namely *Ficus thinning Blume*, *Terminalia schimperiana Hochst*, *Faurea speciosa* Welw, *Echinops sphaerocephalus* L., *Securidaea longepedunculate*, *Lannea schimperi (A. Rich.) Engl., Millettia ferruginea (Hochst. ex Nees) Tander*, and *Guizotia scabra* were harvested from forest and 25 % (5/20) from home garden or farmland. The remaining 30 % (6/20) namely *Croton macrostachyus Del*., *Agave sisalana Perrine ex Engeln*., *Ocimum lamiifolium Hochst*, *Vernonia amygdalina Del*., *Momordica foetida Schumach*, and *Nicotiana tabacum*, were collected from both forest and home garden settings.

**Table 2:** Ethnobotanical data was collected from three local kebeles-Lilisasa Dimtu, Karsa Dako and Mada Jalala-in Arjo Gudatu District, Oromia region, Ethiopia.

Family Name	Scientific Name	Vernacular Name (Afan Oromo)	Voucher No.	Freq. of Citation (%)	Origin	Growth Form	Insects Reported by Participants
Polygalaceae	*Securidaea longepedunculata*	Tebenaye	L04	31 (12.3 %)	Wild	Tree	Mosquitoes, houseflies, ticks, snails
Asteraceae	*Echinops sphaerocephalus*	Kebericho	L02	28 (11.1 %)	Wild	Herb	Mosquitoes
Proteaceae	*Faurea speciose*	Garree	L06	21 (8.3 %)	Wild	Tree	Mosquitoes, cockroaches
Agavaceae	*Agave sisalana*	Kacaa	L08	21 (8.3 %)	Both	Herb	Mosquitoes, ticks
Euphorbiaceae	*Croton macrostachyus*	Bekenisa	L11	19 (7.5 %)	Both	Tree	Mosquitoes
Combretaceae	*Terminalia schimperiana Hochst*	Debeka	L05	19(7.5)	Wild	Tree	Mosquitoes
Acanthaceae	*Justicia schimperiana(hochst.ex Nees) T Ander*	Dhumuga	L10	17(6.7)	Both	Herbs	Mosquitoes
Compositeae	*Guizotia scabra*	Hadaa	L12	15(5.9)	Wild	Herbs	Mosquitoes
Moraceae	*Ficus thinning Blume*	Bifti	L03	15(5.9)	Wild	Tree	Mosquitoes
Cucurbitaceae	*Momodicafoetida schumach*	Imbayoo	L09	12(4.7)	Both	Herbs	Mosquitoes and housefly
Anacardiadceae	*Lannea schimperi(A Rich) Engl.*	Warake	L01	10(3.9)	Wild	Tree	Mosquitoes, houseflies, and cockroaches
Fabaceae	*Millettia ferruginea (Hochst.ex nees) tander*	Sotolo	L07	9(3.5)	Wild	Tree	Mosquitoes, thick, crustaceans, fish, and cockroaches
Brassicaceae	*Lepidium sativum Linn*	Shinfa	L13	8(3.2)	Cultivate	Herbs	Mosquitoes, thick and cockroach
Cruciferae	**Table:** *Brassica nigra Linn. Koch*	Sinafica	L14	6(2.4)	Cultivate	Herbs	Mosquitoes, thick and cockroach
Alliaceae	*Allium sativum Linn.*	Kulubi	L15	6(2.3)	Cultivate	Herb	Mosquitoes
Asteraceae	*Vernonia amygdalina Del.*	Ebicha	L16	5(1.9)	Both	Tree	Mosquitoes
Lamiaceae	*Ocimum lamiifolium Hochst*	Damakase	L17	3(1.2)	Both	Herbs	Mosquitoes
Rutaceae	*Ruta chalepensis L.*	Tenadema	L18	3(1.2)	Cultivate	Herbs	Mosquitoes
Solanaceae	*Nicotiana tabacum*	Tambo	L19	3(1.2)	Both	Herb	Mosquitoes
Verbanaceae	*Lippia adonis*	Kosorota	L20	2(0.7)	Cultivate	Herbs	Mosquitoes and cockroach

Voucher numbers refer to plant specimens collected, identified, and deposited in the National Herbarium of Addis Ababa University, Ethiopia, for reference*.*

#### 3.2.1 Affordable and accessible frequently mentioned plants

*Momordica foetida Schumach (12/12; 100 %) and Echinops sphaerocephalus L. (28/28; 100 %)* were perceived as the most accessible plants followed by *Justicia schimperiana (16/17; 94 %), Croton macrostachyus Del* (17/19; 89 %), and *Millettia ferruginea (Hochst. ex Nees) T. Anders.* (8/9; 88.9 %) (Table 3). *Croton macrostachyus Del* was mentioned as the most Affordable *(19/19; 100 %),* followed by *Justicia schimperiana (Hochst. ex Nees) T. Anders* (17/17; 100 %), *Agave sisalana Perrine ex Engelm* (20/21; 95.2 %), *Faurea speciosa Welw* (18/21; 85.7 %) and *Millettia ferruginea (Hochst. ex Nees) T. Anders* (9/9; 100 %). On the other hand*, Lannea schimperi (A. Rich) Engl.* was perceived as the least accessible (6/10; 60 %) and the least affordable (7/10; 70 %). (See Table 4, Table 5.)

**Table 3:** Perceived accessibility and affordability of the most frequently cited insect-repellent and insecticidal plants, based on responses from participants in Lalisa Dimtu, Karsa Dako, and Mada Jalala kebeles, Arjo Gudatu district, Oromia region, Ethiopia.

Plant species	Accessible		Affordable	
	Yes, n/N (%)	No, n/N(%)	Yes, n/N(%)	No, n/N(%)
*Agave sisalana Perrine ex Engel*	17/21(80.9)	4(19)	20(95)	1(5)
*Croton macrostachyus Del*	17/19(89)	2(10.5)	19(100)	0
*Echinops sphaerocephalus L.*	28/28(100)	0	28(100)	0
*Faurea speciosa Welw.*	14/21(66.6)	7(33.3)	18(85.7)	3(14.3)
*Ficus thonningii Blume*	11/15(73)	4(27)	12(80)	3(20)
*Guizotia scabla*	10/15(66.6)	5(33.3)	12(80)	3(20)
*Justicia schimperiana (hochst.ex Nees) T Ander*	16/17(94)	1(5.8)	17/17 (100)	0
*Lannea schimperi(A Rich) Engl.*	6/10(60)	4(40)	7(70)	3(30)
*Millettia ferruginea (Hochst.ex nees) tander*	8/9(88.9)	1(11)	9(100)	0
*Momodicafoetida schumach*	12/12(100)	0	12(100 %	0
*Securidaea longepedunculate*	20/31(64.5)	11(35.5)	25(80.6)	6(19.4)
*Terminalia schimperiana Hochst*	15/19(78.9)	4(21)	16(84.2)	3(15)

**Table 4:** Attitudes of Participants in Lalisa Dimtu, Karsa Dako, and Mada Jalala Kebeles, and Arjo Gudatu District, Oromia Region, Ethiopia, Toward the Use of Plants for Mosquito Control.

Variable	Strongly agree 253(%)	Agree 253(%)	Unsure 253(%)	Disagree 253(%)	Strongly disagree 253(%)
Efficacy	46(18.2)	154 (60.8)	34(13.4)	19(7.5)	0
Safety	56(22)	144(56.9)	38(15)	15(5.9)	0
Acceptance	75(29.6)	125(49.4)	53(20.9)	0	0
Modernize	57(22.5)	143(56.5)	47(18.5)	6(2.3)	0
Recommendation	54(21.3)	146(57.7)	44(17.4)	9(3.5)	0

**Table 5:** Chi-square analysis of knowledge, attitudes, and practices of Lalisa Dimtu, Karsa Dako, and Mada Jalala Kebele, Arjo Gudatu district, about insecticidal plants.

Variable	Variable category	Number responders	Knowledge of mosquitocidal plants		χ^2^, df, p_ value	Attitude on plants as mosquitocidal		χ^2^, df, p_ value	Practice of mosquitocidal plants		χ^2^, df, p_ value
			No	Yes		No	Yes		No	Yes	
Sex	Male	269	79	186	X^2^ = 13.2 df = 1 *P* = 0.001	29	145	X^2^ = 0.26 df = 1 P = 0.611	130	138	X^2^ = 4.5 df = 1, *P* = 0.034
	Female	129	62	67		12	55		77	52	
Income	<500	90	42	46	X^2^ = 19.9 df = 4 P = 0.001	8	37	X^2^ = 3.01 df = 4 *p* = 0.55	58	32	X^2^ = 18.269, df = 4, *p* = 0.001
	501–700	63	32	31		8	20		40	23	
	701–900	67	16	51		6	41		23	44	
	901–1100	91	62	29		9	50		44	46	
	>1100	87	63	22		10	52		42	45	
Kebele	Mada jalala	150	37	110	X^2^ = 12.3 df = 2 *P* = 0.002	19	88	X^2^ = 0.12 df = 2 *P* = 0.94	65	84	X^2^ = 8.051 df = 2 *P* = 0.018
	Lalisa dimtu	144	59	84		13	67		79	65	
	Karsa dako	104	45	59		9	45		63	41	
Age	19–30	21	6	15	X^2^ = 9.1 df = 4 P = 0.058	6	6	X2 = 10.7 df = 4, *p* = 0.031	13	8	X2 = 7.2 df = 4, *p* = 0.125
	31–40	61	25	36		8	25		37	24	
	41–50	121	49	70		6	63		63	58	
	51–60	124	46	76		12	63		66	57	
	>60	71	15	56		9	43		28	43	

### 3.3 Attitudes of respondents toward using plants for mosquito control

Of the respondents who were aware that plants are used for mosquito control, 79 % (200/253) were positive about their use. Around 60.8 % of those surveyed (154/253) agreed that the plants they had mentioned during the interview were effective against mosquitoes. In addition, 57.7 % indicated that they would recommend the use of these plants to others, and a similar ratio (56.9 %, 144) believed that plants were safe to use.

### 3.4 Plant-based mosquito control practices by household

In total, 75 % (190/253) of the respondents had practiced plants for insecticides and repellents. The respondents cited 3, 5, and 6 plant species as adulticidal, larvicidal, and repellent, respectively, and 6 plants were used for both repellent and adulticidal purposes. Plants belonging to the species *Millettia ferruginea* were the most reported plant species for their larvicidal properties. In addition, the species *Securidaea longipedunculata* is the most reported plant for adulticidal and repellent insects.

#### 3.4.1 Plant parts used for the control of mosquitoes

**Fig. 2:**
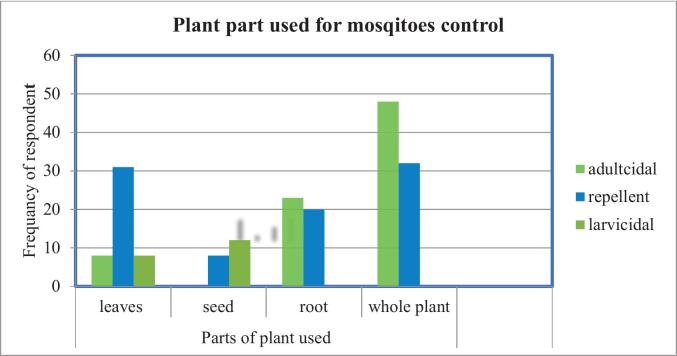
Frequency of respondent for different plant parts used in mosquito control, of Lilisa Dimtu, Karsa Dako and Mada Jalala kebeles-in Arjo Gudatu District, Oromia region, Ethiopia. *n* = 190/253.

As Fig. 2 shows, for adulticidal applications, 25 % (48/190) of respondents reported using the whole plant, followed by 13 % (23/190) who used roots, and 4 % (8/190) who used leaves. For larvicidal applications, seeds were the most commonly reported plant part, at 6 % (12/190), followed by the leaves at 4 % (8/190). When it comes to repellent purposes, leaves were the most commonly used plant part, at 18.4 % (35/190), followed by the whole plant at 14.7 % (28/190), and the root 10.5 % (20/190).

#### 3.4.2 Methods of preparing and using plants- based mosquitoes

**Fig. 3:**
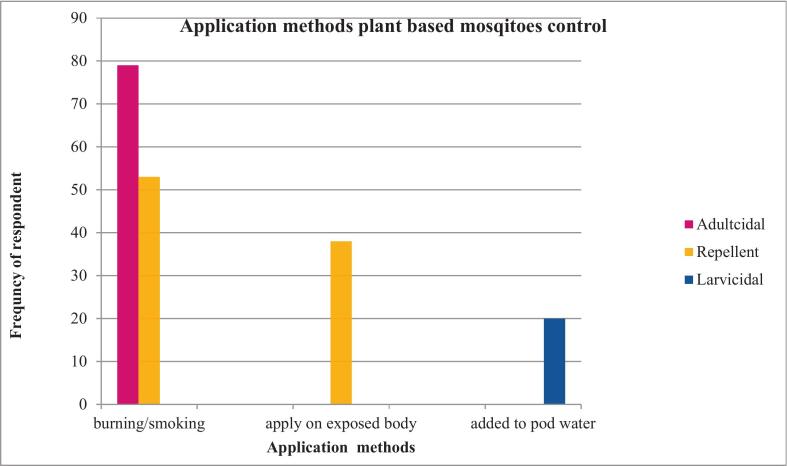
Frequency of respondent for different plant-based application methods used in mosquito control in Lalisa Dimtu, Karsa Dako, and Mada Jalala kebeles, Arjo Gudatu district, Oromia Region, Ethiopia. n = 190/253.

As shown in Fig. 3, Approximately 41.6 % (79/ 190) reported using plants as adulticides by burning or smoking with traditional charcoal stoves, and 27.9 % (53/190) used plants as mosquito repellents. Applying plant to exposed skin as repellents (20 %; 38/190) and adding plant powder to standing water for larvicidal purposes (10 %; 20/190) were also the control strategies.

#### 3.4.3 Timing of plant-based mosquitoes control methods among residents

**Fig. 4:**
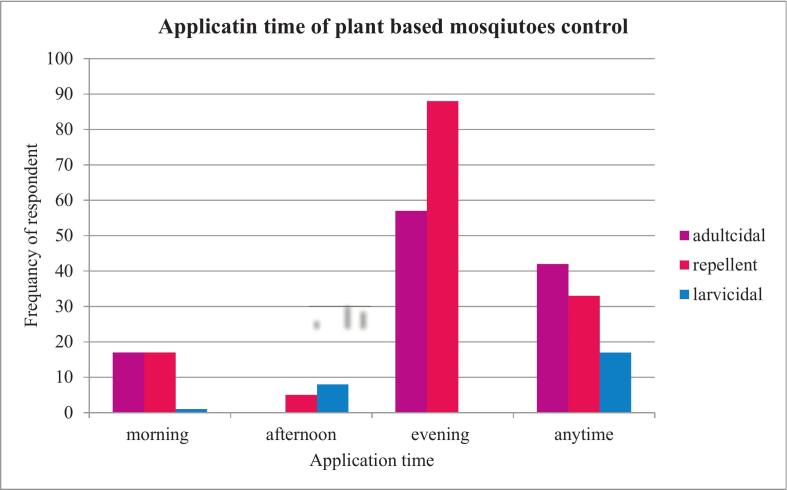
Frequency of respondent for different application times of plant-based mosquito control methods in Lalisa Dimtu, Karsa Dako, and Mada Jalala kebeles, Arjo Gudatu district, Oromia Region, Ethiopia. n = 190/253.

As shown in Fig. 4, Participants used the plants for various activities, including adulticidal purposes, usage peaked in the evening at (30 %), followed by any time (22 %), and in the morning (8.9 %). For repelling mosquitoes using plants, the evening was the most (46.3 %) preferred time followed by any time (17.4 %) and morning time (8.9 %). In contrast, plant application for larvicidal activity was performed predominantly at any time (7.2 %), followed by the afternoon (4.5 %) and the morning (1.5 %).

### 3.5 Associations of socio-demographic factors with knowledge, attitudes, and practices of plant use to control mosquito

The number of males who perceived that plants are used to control mosquitoes was greater than the females (χ^2^ (1) = 13.2, *p* < 0.001). Respondents with low incomes demonstrated higher levels of knowledge compared to those with high incomes (χ^2^ (4) = 19.9, p < 0.001). Furthermore, the Mada Jalala community showed greater awareness than the Lalisa Dimtu and Karsa Dako kebeles communities (χ^2^ (2) = 12.3, *p* < 0.002). In contrast, no significant differences in knowledge were found based on age (χ^2^ (4) = 9.1, *p* = 0.058). Older respondents demonstrated more positive attitudes than their younger counterparts (χ^2^ (1) = 10.7, *p* < 0.031) about mosquitocidal plants. No significant difference was noted between males and females concerning their attitudes (χ^2^ (1) = 0.26, *p* = 0.611). Males were more likely to use plants for vector control than females, and low-income respondents were more inclined to use plants compared to the high-income (χ^2^ (4) = 18.27, p < 0.001). No significant differences in practices were observed by age.

## 4 Discussion

About 64 % (253/398) of the residents in Lalisa Dimtu, Karsa Dako, and Mada Jalala Kebeles (villages) in the Arjo Gudatu District of the East Wollega Zone were aware of the use of locally available plants for the control of insects, most importantly mosquitoes. This was a lower level of community familiarity with insecticidal plants than previous reports from Addis Zemen (97.2 %) (Karunamoorthi et al., 2009a), Jimma zone (83.6 %) (Karunamoorthi et al., 2009b), Western Hararghe Zone (92.1 %) (Karunamoorthi and Husen, 2012), and Tigray region (95 %) (Kidane et al., 2013). This variation could be attributed to differences in the accessibility and availability of modern tools in the kebeles of the district. However, there is still a lack of cultural knowledge about mosquito control plants. Therefore, it is imperative to conduct more ethnobotanical surveys to document and preserve Indigenous knowledge adequately.

Seventy-two percent of respondents were aware of the presence of repellent plants in various epidemic-prone areas of Ethiopia (Karunamoorthi and Hailu, 2014). Studies that were conducted on two kebeles of Sisiga woreda, Karsa Mojo (78.3 %) and Mada Jalala (74.4 %), reported that they knew traditional medicinal plants that are used for malaria prevention and treatment in their localities (Kenea, 2015). The findings from the current study are in line with those reported in previous research, although no formal statistical comparison was made. Most of the participants (60 %) reported receiving their cultural knowledge from a comprehensive society, while 40 % learned from family members. This valuable knowledge, including traditional practice for mosquito control, is passed orally through generations. Such oral transfers play an important role in maintaining domestic health services in rural areas where access to modern medical services is limited.

In total, twenty plants were cited as effective larvicidal, adulticidal, and repellent agents against insects, of which the twelve most frequently cited were *Croton macrostachyus Del, Ficus thonningii Blume, Terminalia schimperiana Hochst Justicia schimperiana (hochst. ex Nees) T Ander, Faurea speciosa Welw., Agave sisalana Perrine ex Engel, Echinops sphaerocephalus L., Securidaea longepedunculate, Lannea schimperi (A Rich) Engl., Millettia ferruginea (Hochst. ex nees) tander, Momodicafoetida schumach*, and *Guizotia scabla*. These plants represent rich reservoirs of traditional knowledge and practical solutions for insect control by the residents. Some plants, such as *Securidaca Longpedunculata*, *Faureaks Speciosa* and *Lanna Schimperi*, are less often reported outside African and may represent specific ethnic knowledge for this region (Gabriel et al., 2015; Chhabra et al., 1987) Opposite *sativam (garlic*), *Nicotiana Tabakam (tobacco*), *Okimum Lamifolium*, *Roota Shailepensis* and *Lapidium Sativam* are widely distributed and cited for their insect discipline or pesticides in African and elsewhere (Zhan et al., 2009; Akumefula et al., 2014).

*Securidaca longepedunculata* is a well-known plant in African ethnomedicine, traditionally used to repel mosquitoes by burning its roots, often in combination with other plants. This practice is common across various African communities, where the plant is also used to treat malaria, fever, and related ailments (Mongalo et al., 2015). Scientific studies have confirmed its mosquito-repellent properties; both crude extracts and essential oils from the roots have shown significant repellency against *Anopheles gambiae* and *Culex quinquefasciatus*. Notably, at concentrations of 0.3 and 0.5 mg/ml, they outperformed the synthetic repellent N, Ndiethyl-m-toluamide (DEET) in laboratory tests (Ogban et al., 2020).

The primary volatile compound responsible for the repellent effect of *Securidaca longepedunculata* is methyl salicylate, which can make up to 90 % of its volatile emissions. Methyl salicylate is known for its strong insect-repellent properties and also functions as a common plant stress signal (Jayasekara et al., 2005).

*Milletia ferruginea* is traditionally used in Ethiopia for mosquito control, particularly in regions like Arjo Gudatu District in Oromia. respondents reported using the plant especially its leaf parts for its larvicidal effects. Scientific studies have supported these traditional uses, confirming the mosquitocidal properties of *Millettia ferruginea's.* for instance, laboratory evaluations have shown that essential oils and extracts from *Millettia ferruginea* possess strong larvicidal, pupicidal, and adulticidal effects against *Anopheles gambiae*, a major malaria vector at a concentration of 100 ppm, the essential oil caused 100 % mortality in second instar larvae and pupae after 72 h. For adult mosquitoes, a 10 % essential oil concentration resulted in 95.66 % mortality after just one hour of exposure, with a rapid knockdown effect (median knockdown time of 7.34 min) (Jemberie et al., 2019).

*Echinops sphaerocephalus* is among the traditionally used plants for mosquito control in Ethiopia, primarily by burning it to produce smoke that repels and kills adult mosquitoes and rich in bioactive secondary metabolites, especially thiophenes and terpenoids, which are believed to contribute to its insecticidal and repellent properties. In addition scientific studies on related *Echinops* species have shown significant mosquito repellent and larvicidal activity. For example, smoke from Echinops leaves (notably E. kebericho, a related species) achieved over 92 % repellency against *Anopheles arabiensis* (Bitew and Hymete, 2019).

*Croton Macrosticions* and *Vernia Amigdalina* have also been examined for larvicidal and ovicidal activities, which support their traditional applications (Karunamoorthi and Ilango, 2010; Okolie et al., 2023). Similarly, essential oils from *Ocimum Lamiifolium* and the *Chalepensis* route have shown strong distorted properties (Alouani et al., 2017). In addition several studies have confirmed that extracts from the leaves, fruits, and flowers of *Momordica charantia* possess significant larvicidal activity against major mosquito vectors, including *Aedes aegypti*, *Anopheles stephensi*, and *Culex quinquefasciatus* (Jayasekara et al., 2005).

*Ficus thonningii Blume, which is one of the species quoted in this study, contained a countless of bioactive compounds including alkaloids, terpenoids, flavonoids, tannins, saponins, and essential oils.*

The secondary metabolites were said to contain antimicrobial and even insecticidal activity, which can be explained by its application in conventional vector control (Debella et al., 2007). Locally, the plant is utilized primarily by locals for Arjo Gudatu District, for a repellent and killing against mosquitoes. Although other constituents and essential oils of *Ficus thonningii Blume* have been found to possess biological activities in previous studies, there is limited or no knowledge of the chemical composition of this species in our area. Furthermore, some of the other species noted in this study *Momordica charantia* and *Echinops sphaerocephalusare* also indicated in the study to have similar bioactive compounds, whose insecticidal potential remains to be fully investigated. Further phytochemical and entomological investigation is needed to validate these traditional claims and to assess their promise for integrated vector management.

Some plants such as *Faurea speciose* and *Guizotia Scabra* are limited evidence, indicating further medical verification requirements. Overall, the findings suggest that some plants have written bio -activity, others represent promising candidates for entomological studies and natural product growth of specially limited advance reports.

Previous studies in Ethiopia indicated varying numbers of plants that are utilized for vector control. For instance, eight plants were reported as insecticides and repellents in Jabitehnan District (Berhanu et al., 2006), and 19 plant species as repellents in Seweyna District, Bale Zone (Shibeshi et al., 2024). In Tanzania, *Azadirachta indica*, *Annona* spp., *Citrus* spp8.3 %, *Ocimum* spp., *Anacadium occidentale*7.1 %, 5.4 % *Mangifera indica*, *Psidium* spp. and *Cocos* lucifer were reported as by communities (Innocent et al., 2014). Close to 74 % of respondents reported that the insecticidal plants are easy to access, and 89 % stated that the plants are affordable, underscoring their significant impact on biodiversity and other ecological factors. This finding is consistent with a previous report (2009) (Karunamoorthi et al., 2009b), in Ethiopia, where insecticidal plants are likely due to the country's rich biodiversity and distinctive ecological environment (Fashing et al., 2022). Their accessibility and affordability suggest the potential for sustainable vector control using locally sourced and culturally accepted strategies. Public health initiatives can utilize this advantage by promoting the use of indigenous insecticidal plants, supporting community-based harvesting practices, and ensuring that resources are preserved and sustainably managed.

Although access and ability data were collected for all plants, the decision to introduce only the most cited species could show bias in the perception of extensive accessibility. Less frequently mentioned plants may still be locally important or accessible, but summary tables are small in tables.

While our data on access and ability are limitations, they provide a valuable starting point for future research. More detailed financial studies are necessary to assess the right cost and availability of these facilities and understand the obstacles that prevent widespread adoption.

The respondents revealed a strong positive attitude toward the traditionally claimed insecticidal plants, of which 79 % expressed satisfaction and more than 84 % acknowledged their effectiveness. This suggests that insecticidal plants are well known in the community as effective, safe, and acceptable. A small percentage expressed their negative opinion toward the plants, possibly influenced by factors including religion and cultural practices. For instance, certain religious doctrines may discourage the use of specific plants or methods, viewing them as incompatible with spiritual or cultural values. Cultural preferences for alternative methods, such as synthetic insecticides or other traditional practices, might also play a role in shaping these negative attitudes (Walker et al., 2014).

Approximately 75 % of respondents practiced using plants for insecticidal and repellent purposes, indicating a strong reliance on traditional methods for vector control. This high percentage reflects widespread knowledge and trust in botanical solutions, possibly due to their accessibility, cost-effectiveness, and historical efficacy. Approximately 91.8 % of the respondents reported that they used repellent plants in various epidemic-prone areas of Ethiopia (Karunamoorthi and Hailu, 2014). This is higher compared to what is presented in the current study. The excessive use of repellent plants in the above research may be attributed to limited access to health facilities, the lack of government control and preventive interventions, and the high endemicity of malaria in the region, which may compel communities to depend increasingly on traditional and self-protection methods. In Tanzania, approximately 22.8 % of respondents reported familiarity with traditional insecticidal practices (Innocent et al., 2014). In contrast, our research indicated that a greater percentage of respondents were familiar with and practiced insecticidal plant methods. This difference may be due to variations in sample sizes and demographic characteristics between the studies.

Approximately 34 % and 40 % of people burned and smoked for repellent and adulticidal activities, respectively. Most of the plants in the community used their whole parts, but only a few plants used their roots and leaves. But the people used them.

In Guinea-Bissau, 55 % of people burn plants to repel mosquitoes (Palsson and Jaenson, 1999), and up to 100 % of Kenyans burn plants to repel mosquitoes (Seyoum et al., 2003), which is higher than the current result. The other finding in the Tigrayi region was that approximately 73 % of respondents smoked by burning a particular plant part (Kidane et al., 2013). Smoking was a common practice among the majority of the community, likely because burning or smoking plants releases volatile compounds that can deter or kill mosquitoes.

In the present study, respondents identified several plants used for adulticidal and repellent purposes through smoking, such as *Agave sisalana Perrine ex Engel*, *Echinops* sp., *Securidaea longipedunculata*, *Terminalia schimperiana Hochst*, *Justicia schimperiana (Hochst. ex Nees) T*. *Ander*, *Faurea speciosa Welw*., and *Lannea schimperi (A. Rich) Engl*. Such an activity, entrenched in the local culture, provides rapid and specific control of mosquitoes. Some of the plants quoted by the respondents as being used regularly for smoky mosquito control include *Croton macrostachyus Del*., *Agave sisalana Perrine ex Enge*l., *Echinops* sp., *Securidaca longepedunculata*, *Terminalia schimperiana Hochst.*, *Justicia schimperiana (Hochst. ex Nees) T. Anders*., *Faurea speciosa Welw*., and *Lannea schimperi (A. Rich.) Engl*.

The respondents reported that they applied crushed *Momordica foetida, Guizotia scabra, Vernonia amygdalina,* and *Ocimum lamiifolium,* directly on their skin to prevent insect bites, especially before bedtime and during farming. This practice targeted mosquitoes and other harmful insects. Similar procedures were observed in the West Gojjam district of Jabitehnan, where plant infusions were applied to exposed body parts for insect-repellent purposes (Berhanu et al., 2006). In another study conducted in the country, 7.53 % of repellent plant extracts were sprayed after being crushed or ground. Additionally, 0.86 % of respondents reported hanging crushed plants, and 0.11 % mentioned sprinkling leaves of repellent plants on the ground (Karunamoorthi et al., 2009b). These results highlight the diverse applications employed by different communities to utilize plant-based insect repellents. Crushing and applying plants directly to the skin is a common and effective practice for immediate protection while spraying and other methods involve a variety of traditional approaches to insect repellency. The varying percentages also suggest differing levels of reliance on and effectiveness of these methods across the regions and communities.

The use of plant residues as a foliar treatment to kill mosquito larvae is an efficient, environmentally friendly, and culturally significant method of control. The plants used *Lippia adoensis*, *Lepidium sativum*, *Brassica nigra*, *Millettia ferruginea*, and *Ruta chalepensis* reflect the wealth of ethnobotanical knowledge available among the community and their capacity to harness natural materials for mosquito control. While scientific validation of the efficacy of these practices is required, this traditional knowledge provides a good foundation for formulating natural insecticide products and for integrating traditional methods with modern vector control practices.

The study revealed a significant relationship between respondents' income and sex, as well as their knowledge of insecticidal plants, but no correlation with age. Male respondents exhibited a greater understanding of insecticidal plants compared to their female counterparts. This disparity may stem from men's perceived responsibility to safeguard their families and themselves from illnesses and insect bites. It is important to note that the majority of survey participants were men. However, these findings stand in contrast to those reported by Karunamoorthi and Hailu (2014) (Karunamoorthi and Hailu, 2014). The study was conducted in western Jamaica and gender was an important factor in respondents' knowledge (Alobuia et al., 2016). The results of the study support the current finding that male respondents were more knowledgeable than females.

These results are comparable to those of previous studies conducted in Ethiopia in endemic areas on insect-repellent plants, where sex and average monthly income were significantly associated (Karunamoorthi and Hailu, 2014). However, insecticide use was not significantly associated with age but was significantly associated with the monthly income and gender of the respondent. Interestingly, the study also found that insecticide use was significantly associated with gender and monthly income, but not with age. This divergence indicates that economic factors and gender roles may play a crucial role in decision-making about insecticide use. For example, higher income could allow better access to commercial insecticides, which are often more expensive than natural alternatives. In addition, gender dynamics could influence household responsibilities and exposure to insects, which in turn affect insecticide use patterns.

The lack of a significant correlation between insecticide use and age suggests that age-related factors, such as generational knowledge, may not strongly influence the decision to use insecticides. This could suggest that the acceptance or need for insecticides is greater across all age groups within the community, possibly due to the efficacy and convenience of these products compared to natural methods.

## 5 Conclusion and recommendations

About 20 plant species were identified to be used by the locals for insect control, with leaves and whole plants being the most exploited plant parts. The most frequent use was by smoking. The practice reflects a strong foundation for traditional ecological knowledge, and society is aware and has a positive attitude toward indigenous plant-based mosquito control methods. However, despite this, we recommend more laboratory and field-based studies to examine the bioactivity and safety of these plants. Due to their scope, efficacy, and cultural acceptability, authentic plant-based approaches can be a long-term alternative in integrated mosquito control programs, if supported with strong scientific evidence and collaboration with the local public health policy.

## CRediT authorship contribution statement

**Lensa Tesfaye:** Writing – original draft, Writing – review & editing. **Esayas Aklilu:** Writing – review & editing. **Ketema Tolossa:** Writing – review & editing. **Abebe Animut:** Writing – review & editing.

## Declaration of competing interest

The authors declare that there is no conflict of interest regarding the publication of this paper.

## Data Availability

None declared.

## Appendix A Supplementary data

Supplementary material Image 1
