# Supplementary material for: Community knowledge, attitudes, and practices regarding the use of plants for mosquito control: The case of Arjo Gudatu District, East Wollega Zone, Oromia Regional State, Ethiopia
Source: Parasite Epidemiol Control. 2025 Nov 4;31:e00463. doi: 10.1016/j.parepi.2025.e00463 (PMC12639579; doi:10.1016/j.parepi.2025.e00463)
Supplement: Supplementary file 1 — Supplementary material [file mmc1.docx]

**Appendix - I**

**Declaration**

We, the authors of this manuscript, hereby declare the following regarding ethics and funding:

**Ethics Declaration:**

- This research was conducted by the Declaration of Helsinki.
- All human participants in this study provided their informed consent to participate.
- Ethical approval for this study was obtained from the Aklilu Lemma Institute of Pathobiology Research Ethics Review Committee, including members Prof. Birhanu Erko (chairperson) and Dr. Alemtsay Teka, (secretary) with the approval number (Ref. No. ALIPB IRERC/108/2015/23). Ethical clearance documentation has been included in the manuscript.
- This research did not involve the use of animals or animal tissues

**Human Ethics and Consent to Participate Declarations:**

- All necessary declarations regarding human ethics and consent to participate have been included in the manuscript.

**Consent for Publication:**

Not applicable.

**Competing Interests:**

The authors declare that they have no competing interests.

**Funding:**

This research received no specific grant from any funding agency in the public, commercial, or not-for-profit sectors.

**Authors' Contributions:**

Lensa Tesfaye - wrote the main text of the manuscript

Abebe Animut, Esayas Aklilu, and Ketema Tolossa - reviewed the manuscript

**Availability of Data and Materials:**

The materials used in this study are available from the corresponding author upon reasonable request.

Signed,

1: Lensa Tesfaye, corresponding author

2: Abebe Animut, co-author

3: Esayas Aklilu, co-author

4: Ketema Tolossa, co-author

**Consent to participate declarations**

**Title:** Community knowledge, attitudes and practices regarding the use of plants for mosquito control: The case of Arjo Gudatu District, East Wollega Zone, Oromia Regional State, Ethiopia

**Name of Principal Investigator:** Lensa Tesfaye

**Name of Organization:** Aklilu Lemma Institute of Pathobiology, Addis Ababa University

**Name of Proposal:** PhD Dissertation

**Good morning/afternoon,**

My name is _________________________ and I am the data collector for a research study being conducted by a PhD student at Aklilu Lemma Institute of Pathobiology, Addis Ababa University. I am here to collect information on the knowledge, attitudes, and practices of the community in Arjo Gudatu District, East Wollega Zone, Oromia Region, Western Ethiopia, regarding the use of indigenous plants for traditional mosquito control. As part of this study, I would like to ask you some questions.

**Participation Information:**

- Purpose: This study aims to collect data on community knowledge, attitudes, and practices related to the use of native plants for mosquito control.

- Duration: Your participation will take approximately 30 minutes.

- Confidentiality: Your responses will be kept confidential, and your name will not be mentioned in the information collected.

- Voluntary participation: Your participation is completely voluntary, and you have the right to stop participating at any time without consequences.

- Benefits and risks: There is no immediate financial benefit for your participation. However, your contribution will provide valuable information that may benefit the community. There are no risks associated with your participation. Your honest answers are crucial to the success of this study and I sincerely ask for your participation.

Based on the information provided, are you willing to participate in this study?

a) Yes, I agree to participate. (Continue with the interview)

b) No, I do not agree to participate. (Thank the interviewee and end the interview)

Name of the interviewer: ______________________________

Signature of the interviewer: __________________________

Date: _____________________

Thank you for your time and participation

**Contact Information:** If you have any questions or need further clarification, you can contact the principal investigator:

**Name:** Lensa Tesfaye, **Address:** Addis Ababa **Cell phone:** +251-908983288 **Email:** [lensat003@gmail.com](mailto:lensat003@gmail.com)

**Appendix – II**

**Ethnobotanical Survey Questionnaire**

**Community Knowledge, Perceptions, and Use of Indigenous Mosquito-Repellent and Insecticidal Plants**

**Part I: Socio-demographic Information**

| **No** | **Question** | **Response Options** | **Remarks** |
| --- | --- | --- | --- |
| 01 | Sex | 1. Male      2. Female |  |
| 02 | Residence | 1. Urban      2. Rural |  |
| 03 | Age | __________ years |  |
| 04 | Family Size | 1. 1–2      2. 3–4      3. 5–6      4. >6 |  |
| 05 | Occupational Status | 1. Peasant      2. Merchant      3. Civil Servant      4. Student      5. Housewife      6. Daily Laborer      7. Other (specify): ________ |  |
| 06 | Educational Status | 1. Illiterate      2. Read and Write      3. Grade 1–5      4. Grade 6–8      5. Grade 9–10      6. Grade 11–12      7. College and Above |  |
| 07 | Monthly Income (ETB) | _____­________________________ | 1 USD ≈ 53.41 ETB |

**Part II: Indigenous Knowledge and Use of Insecticidal/Repellent Plants**

| **No** | **Question** | **Response Options** | **Remarks** |
| --- | --- | --- | --- |
| 08 | What kind of vector control tools can be used? |  |  |
| 09 | Are you aware of any indigenous plants used as insect repellents or insecticides? | 0. No      1. Yes | If No, skip to Question 9 |
| 010 | If Yes, where did you hear about these plants? | ___________________ |  |
| 011 | Can you list the local names of the plants you know? |  | Open- ended |
| 012 | Are these plants accessible and affordable in your community? | 0. No      1. Yes |  |
| 013 | What is the natural habitat of the insecticidal/repellent plants you know? | 1. Wild    2. Home garden/farm    3. Both | Multiple options may apply |
| 014 | What is the growth form of these plants? | 1. Herb    2. Shrub    3. Tree    4. Climber    5. Other: ______ | Choose all that apply |
| 015 | Against which types of insects are these plants used? | _________________________ | (e.g., mosquitoes, flies, cockroaches) |
| 016 | Do you know how these plants work to repel or kill insects? | 0. No      1. Yes |  |
| 017 | If Yes, please describe the mechanism or method of action. | _________________________ | (e.g., smoke, apply to skin) |

**Part III: Attitudes Toward Indigenous Insecticidal/Repellent Plants**

| No | Statement | Strongly Agree | Agree | Unsure | Disagree | Strongly Disagree |
| --- | --- | --- | --- | --- | --- | --- |
| 017 | Indigenous insecticidal/repellent plants are effective in controlling insects. | ☐ | ☐ | ☐ | ☐ | ☐ |
| 018 | Indigenous insecticidal/repellent plants are safe for humans, animals, and the environment. | ☐ | ☐ | ☐ | ☐ | ☐ |
| 019 | These plants are culturally accepted and commonly used in the community. | ☐ | ☐ | ☐ | ☐ | ☐ |
| 020 | These plants should be developed into modern forms like sprays, creams, or ointments. | ☐ | ☐ | ☐ | ☐ | ☐ |
| 021 | I would recommend the use of indigenous insecticidal/repellent plants to others. | ☐ | ☐ | ☐ | ☐ | ☐ |

**Part IV: Practical Use and Handling of Indigenous Insecticidal/Repellent Plants**

| **No** | **Question** | **Response Options** | **Remarks** |
| --- | --- | --- | --- |
| 022 | Have you ever used indigenous plants for insecticidal purposes (adulticidal or larvicidal purposes)? | 0. No      1. Yes | If No, skip Q32 |
| 023 | If Yes, please list the local names of the plants you have used. |  | Open |
| 024 | Which parts of the plant were used for insecticide purposes? | 1. Leaves    2. Roots    3. Seeds    4. Dermis of roots    5. Bark and resin    6. Whole plant & leaves    7. Peels    8. Fruit peels    9. Other: ________ | Multiple answers allowed |
| 025 | How are these plants processed for insecticidal use? | __________________________ | Open-ended |
| 026 | How are these plants applied for insecticidal use? | __________________________ | Open-ended |
| 027 | Have you ever used indigenous plants for repellent purposes? | 0. No      1. Yes |  |
| 028 | If Yes, please list the local names of the plants you have used. |  |  |
| 029 | Which parts of the plant were used for repellent purposes? | (Same options as Q33) | Multiple answers allowed |
| 030 | Do you use fresh or dried plant materials for repellent use? | 0. Fresh      1. Dried | Consider allowing both |
| 031 | How are these plants processed for repellent use? |  | Open-ended |
| 032 | How are these plants applied for repellent purposes? | __________________________ | Open-ended |
| 033 | Which body parts are the repellent plants typically applied to? | __________________________ | Open-ended |
| 034 | At what time of the day do you use these plants? | a) Morning    b) Afternoon    c) Evening    d) Night | Multiple choices allowed |
| 035 | How many times per day do you use these plants for insecticidal/repellent purposes? | __________________________ | Numeric |
|  |  |  |  |
